# P-2349. Clinical and genomic epidemiology of coxsackievirus A21 and enterovirus D68 in homeless shelters, King County, Washington, 2019-2021

**DOI:** 10.1093/ofid/ofae631.2501

**Published:** 2025-01-29

**Authors:** Sarah N Cox, Amanda M Casto, Nicholas M Franko, Eric J Chow, Peter D Han, Luis Gamboa, Brian A Pfau, Hong Xie, Kevin Kong, Jaydee Sereewit, Melissa A Rolfes, Emily Mosites, Timothy M Uyeki, Alexander L Greninger, Marco Carone, Mi-Hyun M Shim, Trevor Bedford, Jay Shendure, Michael J Boeckh, Janet A Englund, Lea Starita, Pavitra Roychoudhury, Helen Y Chu

**Affiliations:** University of Washington, Seattle, Washington; University of Washington, Seattle, Washington; University of Washington, Seattle, Washington; Public Health - Seattle & King County, Seattle, Washington; University of Washington, Seattle, Washington; Brotman Baty Institute for Precision Medicine, Seattle, Washington; University of Washington, Seattle, Washington; University of Washington, Seattle, Washington; University of Washington, Seattle, Washington; University of Washington, Seattle, Washington; Centers for Disease Control and Prevention, Atlanta, Georgia; Multnomah County Health Department, Portland, Oregon; Centers for Disease Control and Prevention, Atlanta, Georgia; University of Washington, Seattle, Washington; University of Washington, Seattle, Washington; Public Health - Seattle & King County, Seattle, Washington; Fred Hutchinson Cancer Research Center, Seattle, WA; University of Washington, BBI, HHMI, Allen Discovery Center, Seattle, Washington; Fred Hutchinson Cancer Center, Seattle, WA; Seattle Children’s Hospital, Seattle, Washington; University of Washington, Seattle, Washington; University of Washington, Seattle, Washington; University of Washington, Seattle, Washington

## Abstract

**Background:**

Congregate homeless shelters are disproportionately affected by infectious disease outbreaks. Although outbreaks of SARS-CoV-2 and influenza viruses have been reported in homeless shelters, there are minimal data describing enterovirus transmission among people experiencing homelessness.**Figure 1.** Nasal swab specimens and enterovirus detection in homeless shelters, October 2019 - February 2020, King County, Washington, USA

**Figure:**
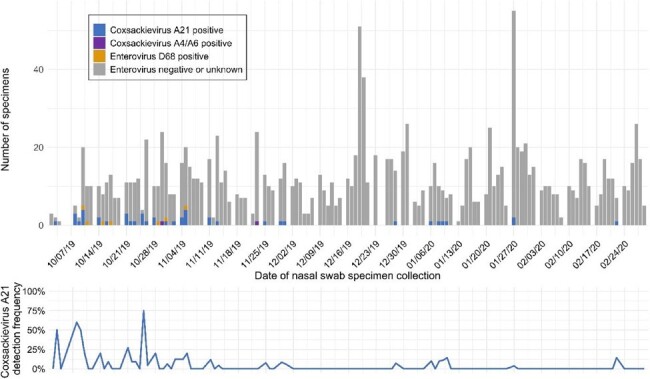
NOTE: No coxsackievirus A21 or enterovirus D68 positive specimens were detected between March 2020 - May 2021

**Methods:**

We describe the epidemiology of enteroviruses across 23 adult and family shelters in King County, Washington from October 2019 through May 2021, using repeated cross-sectional respiratory illness and environmental surveillance. Genomic sequencing was used to describe the molecular diversity of enteroviruses within and across shelter sites.**Figure 2.** Phylogenetic trees of sequenced participant coxsackievirus A21 shelter specimens. A) Tree containing all shelter coxsackievirus A21 and all coxsackievirus A21 genomes deposited in Genbank. Tips representing study specimens are colored according to shelter. Light gray tips represent coxsackievirus A21 genomes downloaded from Genbank. The x-axis represents number of nucleotide changes in the genome relative to a coxsackievirus A21 reference genome (AF465515.1). B) Tree containing all shelter coxsackievirus A21 genomes. Internal nodes with >90% bootstrap support are labeled on tree. C) Tree containing all shelter coxsackievirus A21 genomes with x-axis corresponding to sample collection date.

**Figure:**
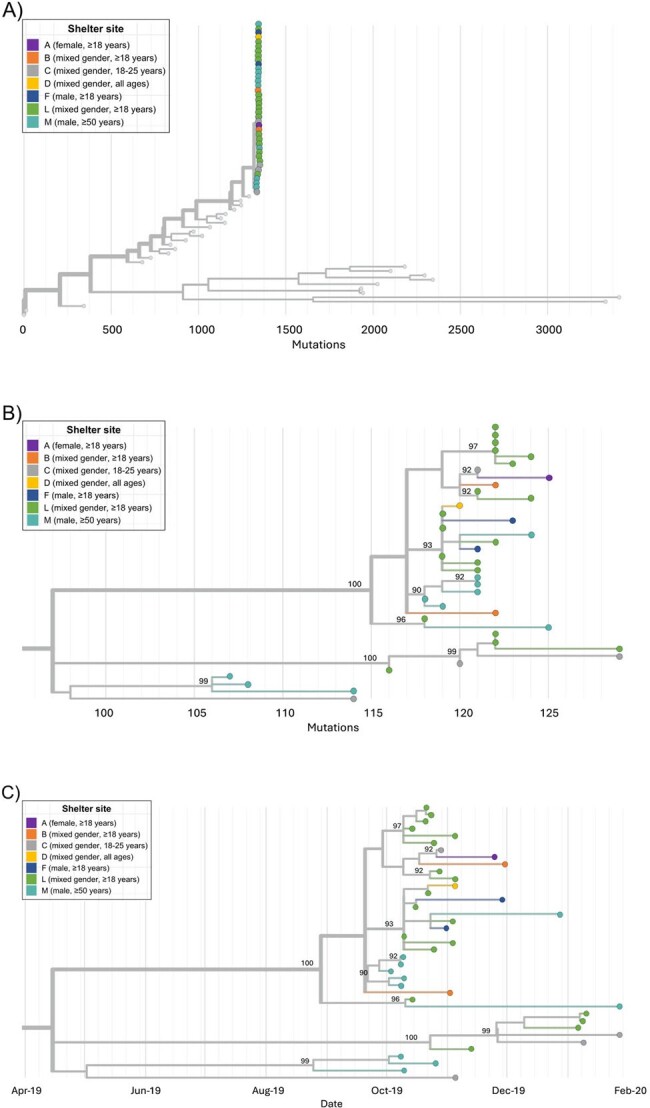

**Results:**

Of 3,281 participants aged ≥3 months, coxsackievirus A21 (CVA21) was identified in 39 adult residents (3.0% detection, 95% CI: 1.9%-4.8%) across seven shelters from October 2019-February 2020 (Figure 1). Enterovirus D68 (EV-D68) was identified in five adult residents in two shelters October-November 2019. The most commonly reported signs or symptoms of both CVA21 and EV-D68 included runny nose and cough. Of 812 environmental samples, one was EV-D68-positive and five were CVA21-positive. Other enteroviruses detected among residents, but not in environmental samples, included coxsackievirus A6/A4 (n=3 children). No enteroviruses were detected from April 2020-May 2021. Closely related CVA21 and EV-D68 cases occurred in some shelters (Figures 2-3). Some shelters also hosted multiple CVA21 lineages (Figure 2).

Figure 3. Phylogenetic tree of sequenced participant enterovirus D68 shelter specimens. Tips representing study specimens are colored according to shelter. Light gray tips represent enterovirus D68 genomes downloaded from Genbank. The inset shows a detailed view of the relationship among the study genomes. The x-axis represents the number of nucleotide changes in the genome relative to an enterovirus D68 reference genome (NC_038308.1).

**Figure:**
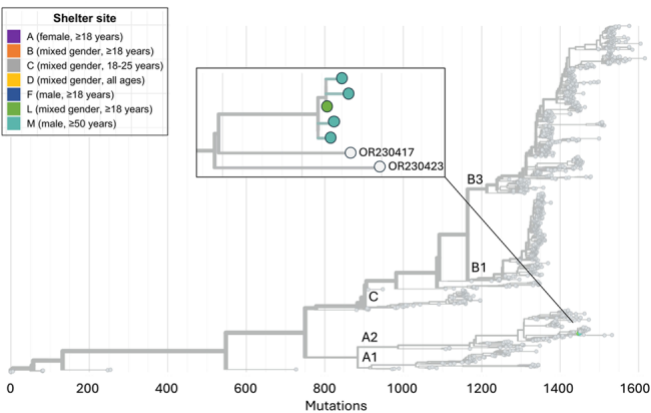

**Conclusion:**

This study provides critical information on clinical features and transmission patterns of enteroviruses in congregate homeless shelters. Surveillance of enteroviruses in shelters and other congregate settings may be warranted for early detection and implementation of control measures to reduce outbreaks.

**Disclosures:**

Eric J. Chow, MD, MS, MPH, IDWeek: Travel Grant|Providence Regional Health Everett: Honoraria Michael J. Boeckh, MD PhD, Allovir: Advisor/Consultant|Allovir: Grant/Research Support|AstraZeneca: Advisor/Consultant|AstraZeneca: Grant/Research Support|Merck: Advisor/Consultant|Merck: Grant/Research Support|Moderna: Advisor/Consultant|Moderna: Grant/Research Support|Symbio: Advisor/Consultant Janet A. Englund, MD, Abbvie: Advisor/Consultant|AstraZeneca: Advisor/Consultant|AstraZeneca: Grant/Research Support|GlaxoSmithKline: Advisor/Consultant|GlaxoSmithKline: Grant/Research Support|Meissa Vaccines: Advisor/Consultant|Merck: Advisor/Consultant|Pfizer: Board Member|Pfizer: Grant/Research Support|Pfizer: Speaker at meeting|SanofiPasteur: Advisor/Consultant|Shinogi: Advisor/Consultant Helen Y. Chu, MD, MPH, Abbvie: Advisor/Consultant|Merck: Advisor/Consultant|Vir: Advisor/Consultant

